# Inequalities in utilization of maternal health services in Ethiopia: evidence from the PMA Ethiopia longitudinal survey

**DOI:** 10.3389/fpubh.2024.1431159

**Published:** 2025-01-07

**Authors:** Asebe Hagos, Misganaw Guadie Tiruneh, Melak Jejaw, Kaleb Assegid Demissie, Lemlem Daniel Baffa, Demiss Mulatu Geberu, Getachew Teshale, Tesfahun Zemene Tafere

**Affiliations:** ^1^Department of Health Systems and Policy, Institute of Public Health, College of Medicine and Health Science, University of Gondar, Gondar, Ethiopia; ^2^Department of Human Nutrition, Institute of Public Health, College of Medicine and Health Sciences, University of Gondar, Gondar, Ethiopia

**Keywords:** inequality, inequity, maternal health, antenatal care, skilled birth attendant, postnatal care, HEAT Plus, Ethiopia

## Abstract

**Background:**

Previous studies documented the existence of substantial inequalities in the utilization of maternal health services across different population subgroups in Ethiopia. Regularly monitoring the state of inequality could enhance efforts to address health inequality in the utilization of maternal health services. Therefore, this study aimed to measure the level of inequalities in the utilization of maternal health services in Ethiopia.

**Method:**

The study used data from the Performance Monitoring for Action Ethiopia (PMA Ethiopia) dataset. Longitudinal data was collected from a weighted sample of 1966 postpartum women using multistage stratified cluster sampling techniques between November 2021 to October 2022. We assessed inequality in maternal health services using three indicators: antenatal care four (ANC), skilled birth attendants (SBA), and postnatal care (PNC). Age, economic status, education level, place of residence, and subnational regions were used as dimensions for measuring inequality. The analysis was conducted using Health Equity Assessment Toolkit Plus (HEAT Plus) software. We computed the summary measure of health inequality: Difference (D), Ratio (R), Population Attributable Risk (PAR), and Population Attributable Fraction (PAF).

**Result:**

The simple summary measures of inequality difference (D) reported a high level of inequality in the utilization of maternal health services in ANC four, SBA, and PNC across economic, education, residence, and subnational regions. The difference (D) in maternal health service utilization between advantaged and disadvantaged population groups exceeded 20 percentage points in all four dimensions of inequality for the three maternal health indicators. Similarly, the complex summary measures of inequality (PAR and PAF) also showed high levels of inequality in the utilization of ANC four, SBA, and PNC across all four dimensions of inequality. However, there was no age-related inequality in the use of maternal health services.

**Conclusion:**

A high level of socioeconomic and geographic area related inequality was observed in the utilization of ANC four, SBA, and PNC services in Ethiopia. Women from socioeconomically disadvantaged subgroups and women from disadvantaged geographic areas significantly lagged behind in the utilization of maternal health services. Therefore, implementing targeted interventions for the most disadvantaged groups can help to reduce inequality in accessing maternal health services.

## Background

Globally, maternal mortality was estimated at 223 maternal deaths per 100,000 live births in 2020. In the past 20 years, the maternal mortality rate (MMR) has been reduced by 34.3% worldwide (1). However, the MMR in the Africa region is still a tragedy, with 545 deaths per 100,000 live births. Nearly 70% of maternal deaths were reported in sub-Saharan African countries, followed by central and southern Asia, which accounted for nearly 17% of maternal deaths (1, 2). In the same year, the highest number of maternal deaths was reported in Nigeria (82,000), India (24,000), the Democratic Republic of the Congo (22,000), and Ethiopia (10,000). According to the United Nations Interagency Maternal Mortality Ratio estimation, MMR declined in Ethiopia from 953 in 2000 to 267/100,000 in 2020 (1).

Health inequalities are visible differences in health determinants and outcomes across populations of subgroups on the basis of demographic, geographic, or socioeconomic factors such as age, economic status, education level, place of residence, and sex (3–5). It is a measured difference in health between population subgroups and is one metric used to assess health equity (6). Inequality is present whenever there are variations in health indicators among different subgroups (5). However, not every difference can be labeled as inequity. Health inequity refers that are unnecessary, avoidable and also deemed unfair and unjust (7). The concept of health equity involves promoting justice and eliminating all forms of discrimination (6). Health equity is an essential component of the vision of health as a fundamental human right and health for all, where everyone can attain their full potential for health and well-being (8).

In Ethiopia, pregnancy-related complications such as hemorrhage, obstructed labor, pregnancy-induced hypertension, puerperal sepsis, and unsafe abortion are reported as the main causes of maternal deaths (9, 10). Access to health services like antenatal care, delivery by trained birth attendants, and postpartum care can effectively reduce the occurrence of pregnancy-related complications and avert maternal and newborn deaths (8, 11). However, women with socioeconomic disadvantages are less likely to have access to and use maternal health services during the most critical times of pregnancy and childbirth (12). Ethiopia has significantly reduced maternal mortality over the last two decades, but the country still accounts for 3.6% of global maternal deaths (1). These maternal deaths were more common among women with lower socioeconomic status and those living in rural areas (13). The high number of maternal deaths reflects inequalities in access to quality health services and highlights the gap between advantaged and disadvantaged women (14).

In Ethiopia, inadequate coverage and a high level of inequalities in the utilization of maternal health services pose significant challenges to attaining the Sustainable Development Goals. According to the latest 2019 Mini Ethiopia Demographic and Health Survey (MEDHS) report, maternal health service indicators remain below national and global targets (15–17). The survey revealed that the utilization rates for ANC four, SBA, and PNC services were 43, 50, and 34%, respectively (16). Additionally, the national documents have shown that there is an inequitable distribution of health outcomes and maternal health services across different subgroups of the population (18). The maternal health indicators vary greatly based on geographic area, gender, disability status, education level, and socioeconomic characteristics (15, 19, 20). Other studies have also reported the existence of substantial inequalities in the utilization of maternal health services among various population subgroups. The use of maternal health services was higher among women with higher economic status and urban residents (17, 19, 21). Women with disadvantaged socioeconomic status were less likely to utilize maternal health services such as ANC, SBA, and PNC (20).

To end preventable maternal deaths, the WHO advises countries to monitor and enhance efforts to address inequities in access to maternal health care to reach vulnerable populations with high quality maternal health services (11). Reducing health inequalities is a key aspect of the Sustainable Development Goals (SDGs) and a top priority for the government of Ethiopia and the WHO (22). In recent years, inequalities in health have received significant attention (23). Several reports have indicated a rising concern over inequality in health outcomes and access to health services in various regions of the world, particularly in low middle-income countries where there is significant inequity in the utilization of health services (6, 24–26).

Addressing inequalities in maternal and child health is crucial for attaining universal health coverage (UHC), protecting human rights, promoting gender equality, avoiding discrimination, and improving social determinants that influence health outcomes (6). Measuring health inequalities (measured differences in health across population subgroups) is a critical step to address health inequities (unjust, unfair, and avoidable health disparities) (27). Countries need to regularly monitor the state of inequality in access to maternal healthcare at the national or subnational level. This involves comparing the level of key maternal indicators across different population segments based on socioeconomic and geographic related characteristics (6, 11, 28). Evidence on the state of inequality can help identify inequity in the utilization of maternal health services and guide the development of policies and implementation of programs to ensure all women have access to the full range of quality maternal health services (5, 6, 23, 29).

However, the level of inequality in the utilization of maternal health services has not been well investigated in Ethiopia using the WHO HEAT Plus. Since inequality monitoring in health is a regular and ongoing practice, no adequate studies have been conducted using latest data in recent years. Furthermore, to the best of our knowledge, no studies have been conducted to measure the level of inequality in the utilization of maternal health services in Ethiopia using the second cohort’s 6-week postpartum survey of the PMA Ethiopia 2021–2023 cohort dataset.

Therefore, this study aimed to measure the level of inequalities in the utilization of maternal health services in Ethiopia. Insight from this study can help policymakers and health managers formulate evidence-based policies and implement effective interventions to reduce inequalities in accessing maternal health service in Ethiopia.

## Materials and methods

### Study settings, design, and data

At the time of data collection, Ethiopia had 10 regions (Tigray, Afar, Amhara, Oromia, Somali, Benishangul-Gumuz, Southern Nations Nationalities and Peoples’ (SNNP), Gambella, Harari, and Sidama) and two city administrations (Addis Ababa and Dire Dawa). Ethiopia’s population is estimated to be 126 million people, making it the second most populous country in Africa (22).

The study used data from the PMA Ethiopia dataset. PMA is a survey project that is designed to generate data on a variety of reproductive, maternal, and newborn indicators (30, 31). Beside the demographic and health survey, it can also serve as an additional data source for monitoring health inequality in reproductive, maternal, and newborn healthcare in Ethiopia. The PMA Ethiopia survey was conducted as the second cohort of a longitudinal study (2021–2023), which enrolled women at baseline and followed them at six weeks, six months, and one year postpartum. For the present analysis, we used data from the second cohort’s 6-week postpartum survey of the PMA Ethiopia panel survey. To get a complete dataset, the six-week follow-up dataset was combined with the panel cohort two baseline dataset (32).

The longitudinal data was collected from Addis Ababa City administration, Amhara, Oromia, and SNNP Region of Ethiopia. The data was collected using multistage stratified cluster sampling techniques. In the first stage, Amhara, Oromia, and the SNNP regions were stratified into urban and rural strata, whereas Addis Ababa City was served as an urban stratum. A total of 162 enumeration areas (EA) were selected with probability proportional to size within strata using a central statistical agency (CSA) sampling frame. At the second stage, 35 households were randomly selected from each enumeration area (31). The second cohort’s 6-week postpartum survey was conducted between November 2021 and October 2022.

### Study population, sample size, and sampling procedure

**Figure 1:**
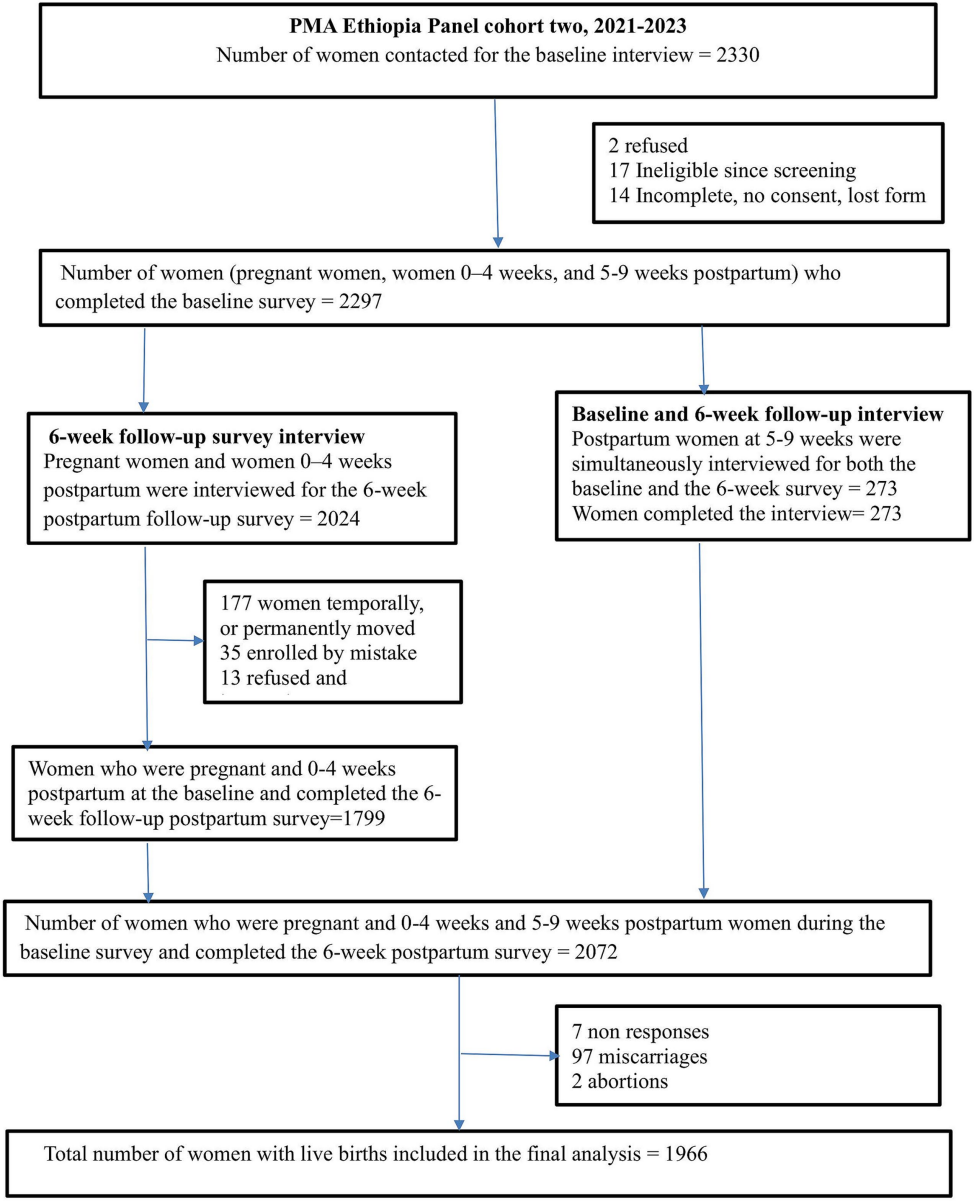
Data extraction and sampling procedure of inequality in the utilization of maternal health service in Ethiopia, PMA 2022.

Women who were 5–9 weeks postpartum at baseline who consented to the baseline survey, women 0–4 weeks postpartum at baseline who consented to the baseline and consented to follow-up, and women pregnant at baseline who consented to the baseline and consented to follow-up were eligible for the 6-week follow-up survey (32, 33). A total weighted sample size of 1966 women was included in the analysis of inequality in the utilization of maternal health services (Figure 1).

### Data collection

For the baseline survey, data collectors collected information on women’s sociodemographic characteristics (age, education, region, parity, residence, marital status, household wealth status, migrant status), fertility preferences, and birth histories. Among women who were pregnant at the time of the baseline survey, information was also collected on estimated gestational age and utilization of maternal health services (31, 33). Similarly, for the six-week postpartum interview, data were collected on key maternal and neonatal health and delivery services, including receipt, timing, and specific components of ANC, delivery-related information, and the receipt of immediate postpartum services for both the mother and child. Data were collected using the Open Data Kit (ODK) system on tablet computers by trained field workers. Prior to data collection, trainings were given for field supervisors and data collectors. The training was focused on a review of the survey protocols, questionnaire content, and interview skills (31).

### Variables and measurement

We used three maternal health indicators: ANC four, SBA, and PNC to measure inequality in the utilization of maternal health services among postpartum women (5, 34). ANC four coverage was defined as the proportion of women who had at least four ANC visits during pregnancy by any provider (skilled health personnel and health extension workers) (16). SBA was defined as the proportion of births attended by skilled health personnel. Skilled health personnel include doctors, nurses, midwives, and health officers (5, 35). Similarly, PNC was computed as the proportion of women who had received at least one postnatal care within 48 h of delivery (33).

The authors used age, economic status, education level, place of residence, and subnational regions as dimensions of inequality measurement. These dimensions are common sources of discrimination, and can be widely applied to inequality studies in low- and middle-income countries (5, 34). Age (categorized as 15–24 years, 25–34 years, and 35–49 years), education level (coded as no education, primary and secondary education, and technical and higher education), place of residence (urban and rural), and subnational region (Addis Ababa City administration, Amhara region, Oromia region, and SNNP).

The wealth index was constructed from a number of indicators that are thought to be correlated with a household’s economic status. Component indicators include, for example, possession of household assets (electricity, television, radio, watch, telephone, refrigerator), types of vehicles, water supply and sanitation (the source of drinking water, type of toilet, sharing of toilet facilities), housing condition (material of the principal floor, walls, roof), and ownership of agricultural land, as well as the type and number of animals owned (36, 37). Principal component analysis was used to construct the wealth index. Finally, the wealth index of the household was ranked in five quintiles: quintile 1 (poorest), quintile 2, quintile 3, quintile 4, and quintile 5 (richest).

### Statistical analysis

The Health Equity Assessment Toolkit (HEAT) and Health Equity Assessment Toolkit (HEAT) Plus are free and open-source software applications that facilitates the assessment of within country health inequalities using disaggregated data (12). HEAT Plus allows users to upload their own databases and assess inequalities at the global, national, or subnational level for a range of (health) indicators and dimensions of inequality (38, 39). Since we prepared a new database from PMA – Ethiopia survey, we used HEAT Plus software Version 5.0, Geneva, WHO,2023 to uploaded the data base and explore, analysis and present inequality in maternal health services. Stata version 17.0 was used for data preparation and analysis.

We employed simple summary measures of health inequality, such as Difference (D) and Ratio (R), along with complex summary measures of health inequality such as Population Attributable Risk (PAR) and Population Attributable Fraction (PAF) to measure the extent of inequality in the utilization of maternal health services. Summary measures of health inequality play a significant role in monitoring inequalities in health. By using disaggregated data, the summary measures quantify the level of inequality in a single number, facilitating comparison over time across various health indicators, programs, and settings (29).

The summary measures express absolute or relative inequality. Absolute inequality measures reflect the magnitude of the difference in health between population subgroups and retain the same unit of measure as the health indicator. Relative inequality measures show the proportional differences in health among subgroups and are unit-less (4, 29). Relative measures are particularly useful when making comparisons between indicators that have different units (4).

The difference (D) is an absolute measure of inequality that shows the difference in the utilization of maternal health services between two population subgroups (in this case, for example, between quintile 5 and quintile 1). D was calculated as: D = y1 − y2, where y1 and y2 indicate the estimates for subgroups 1 (quintile 5) and 2 (quintile 1). A difference value of 0 indicates no inequality (both subgroups have the same level of maternal health service utilization) (29, 39). High inequality denotes an absolute difference of 20 percentage points (pp) or more between two population subgroups. Absolute difference values that fall between these two thresholds (5–20 pp) and less than 5 pp. are considered to be moderate and low inequality in the utilization of maternal health services, respectively (4, 27, 29).

The ratio (R) is a relative measure of inequality that shows the ratio of two population subgroups. R was calculated as: R = y1/y2, where y1 and y2 indicate the estimates for subgroups 1 (quintile 5) and 2 (quintile 1). If there is no inequality, R takes the value one. R takes only positive values (4, 29, 39). High inequality denotes a ratio of ≤0.5 or ≥ 2.0 between two population subgroups. Moderate inequality for ratio values falls either above 0.5 and below 0.9, or above 1.1 and below 2.0 (27). However, the simple measure of inequality (D and R) has two limitations: Firstly, it considers only two subgroups (the poorest and the richest quintile), and ignores the middle quintile (the second, third, and fourth quintile). Secondly, it did not take into account the size of the subgroup population (4, 29). Therefore, we used the complex summary measures (PAR and PAF) in addition to simple summary measures to assess the level of inequality. PAR and PAF take into account all population subgroups based on a weighted sample. They are also calculated for ordered and non-ordered dimensions of inequality.

PAR is an absolute measure of inequality and is calculated as the difference between the estimate for the reference subgroup (yref) and the national average of ANC four, SBA, and PNC (*μ*): PAR = yref-μ, where μ is the national average of ANC four, SBA, and PNC coverage. In this study, yref refers to estimates of ANC four, SBA, and PNC in the: women’s age category 35–49 for age; urban setting for place of residence; technical or higher education for education level; the richest sub-groups for economic status; and Addis Ababa City administrations for subnational regional dimensions. PAF is a relative measure of inequality, and PAF = (PAR/ *μ*)*100. PAR and PAF take positive values for favorable indicators (ANC four, SBA, and PNC coverage). PAR and PAF values of 0 indicate the absence of inequality, and the larger the absolute value, the greater the level of inequality (4, 29, 39).

A 95% uncertainty interval (UI) was calculated around point estimates (Est) as a measure of statistical significance. To indicate the presence of significant inequality in the utilization of maternal health services, the lower bound (LB) and upper bounds (UB) of D and PAR UIs should not include zero. R and PAF inequality exist if UIs do not include one (29, 40, 41).

## Results

### Sociodemographic characteristics of study participants

The study included a total weighted sample of 1966 postpartum women for analysis of inequality in maternal health service utilization. Nearly half (48%) of the women were in the 25–34 age category, and one in three women (31.5%) had no formal education. One in five (19.9%) women belonged to the poorest household. Additionally, three-fourths (75.5%) of the women resided in rural areas, and half (52.3%) of the women were from the Oromia region (Table 1).

**Table 1:** The coverage of maternal health service across different subpopulations in Ethiopia: evidence from PMA-Ethiopia, 2022.

		Weighted frequency (%)	Antenatal care			Skilled birth attendant			Postnatal care		
Dimensions of inequality	Subpopulation	Weighted frequency (%)	Est	LB	UB	Est	LB	UB	Est	LB	UB
Mother’s age at birth	15–24 years	716 (36.4)	42.6	36.6	48.6	65.7	57.2	74.1	44.6	38.1	51.1
	25–34 years	944 (48.0)	47.4	41.4	53.4	61.1	54.4	67.9	41.7	36.5	46.9
	35–49 years	306 (15.6)	38.6	31.6	45.6	53.4	46.2	60.6	39.6	32.2	46.8
Household wealth quintile	Quintile 1 (poorest)	391 (19.9)	31.2	21.6	40.8	32.1	22.4	41.9	21.5	15.1	28.0
	Quintile 2	395(20.1)	34.3	25.3	43.4	42.3	34.1	50.5	25.6	19.3	31.9
	Quintile 3	389(19.8)	36.3	28.1	44.5	56.7	48.4	65.1	37.3	30.2	44.5
	Quintile 4	396(20.1)	50.0	41.5	58.4	80.1	71.9	88.4	55.1	47.1	63.1
	Quintile 5 (richest)	395(20.1)	69.4	62.6	76.3	96.1	93.5	98.6	72.2	65.9	78.5
Mother’s education level	No education	620 (31.5)	33.9	25.7	42.0	41.1	32.9	49.2	27.6	21.9	33.3
	Primary or Secondary education	1,195 (60.8)	45.7	40.5	50.9	67.6	60.6	74.7	45.9	40.1	51.6
	Technical or higher education	151(7.7)	75.9	68.3	83.6	97.8	95.2	100	76.0	70.1	81.9
Place of residence	Rural	1,484 (75.5)	37.2	30.6	43.8	51.3	42.9	59.7	34.6	28.5	40.7
	Urban	482 (24.5)	66.1	59.1	73.2	93.2	89.1	97.2	66.5	60.2	72.9
Subnational region	Addis Ababa City	92(4.7)	72.4	53.7	91.2	99.2	98.1	100	87.1	82	92.3
	Amhara region	413(21.0)	48.9	42.1	55.7	70.7	63.3	78.1	42.5	34.7	50.4
	Oromia region	1,029(52.3)	44.1	35.4	52.8	56.2	45.7	66.7	42.6	34.6	50.6
	SNNP region	432(22.0)	34.4	24.8	44.0	57.6	43.0	72.3	32.4	23.3	41.6

Est, point estimate; LB, lower bound; UB, upper bound.

*SNNP includes Central Ethiopia Regional State, South Ethiopia Regional State, and South West Ethiopia Peoples’ Region.

### Maternal health service utilization across different dimensions

The study showed the coverage of the three maternal health service indicators was low in Ethiopia. The coverage in utilization of SBA was 61.5 (95% CI: 55.07, 68.04), followed by ANC four 44.3 (95% CI:39.1, 49.5) and PNC 42.4 (95% CI: 37.5, 47.3). The coverage of maternal health services varied across the dimensions of inequality within the country. Utilization of maternal health services was higher in women with better economic status. Use of ANC four services was 69.4% in women with the richest wealth status and 31.2% among the poorest. Utilization of SBA was 96.1 and 32.1% from the richest and poorest wealth quintile, respectively. Similarly, PNC was 72.2% for women with the richest household, whereas it was 21.5% for women with the poorest household.

In the present study, we found discrepancies in the utilization of maternal health services based on education level. Women with higher education level were found to be better at utilizing maternal health services. For instance, ANC four and SBA were 98.7 and 75.9% among women with higher education, respectively. On the other hand, the coverage of ANC and SBA was 33.9 and 41.1% among women who had no formal education, respectively.

This study reported urban rural disparities in maternal health service coverage. ANC four service was 37.2% for rural residents and 66.1 for urban residents. Likewise, the use of SBA was 51.3% among rural residents, whereas SBA was 93.2% among rural residents.

**Figure 2:**
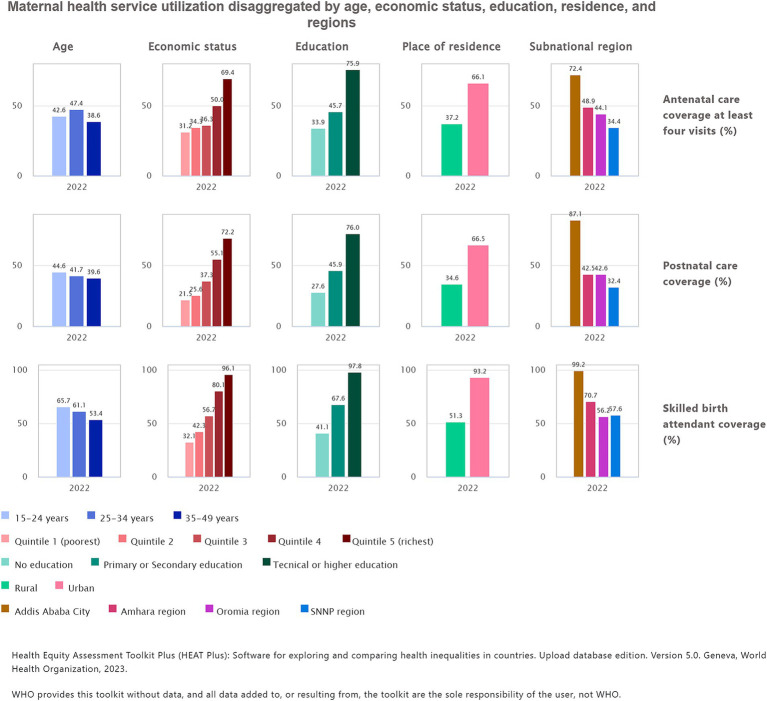
Maternal health service utilization disaggregated by age, economic status, education level, place of residence, and subnational regions in Ethiopia, PMA 2022.

Substantial variation was also reported in the coverage of maternal health service utilization across subnational regions of Ethiopia. For example, higher coverage of ANC four (72.4%), SBA (99.2%), and PNC (87.1%) was reported in Addis Ababa city administration; on the other hand, low coverage of ANC four (34.4%), and PNC (32.4%) was reported in the SNNP region. In addition, low coverage of SBA (56.2%) was reported in the Oromia region of Ethiopia (Table 1; Figure 2).

### Level of inequality in maternal health service utilization

In this study, a high level of economic related inequality was observed in the utilization of maternal health. The simple measure of inequality, D, was 38.3, 64.0, and 50.7 for ANC four, SBA, and PNC, respectively. This indicates the coverage of ANC four, SBA, and PNC in the richest household wealth status was higher by 38.3 pp., 60 pp., and 50.7 pp., respectively, compared with women in the poorest household wealth status. Likewise, the relative measure of inequality R suggests a high level of economic related inequality in the use of maternal health service utilization between the poorest and richest quintiles. The value of R was 2.2 for ANC, 3.0 for SBA, and 3.4 for PNC. This result implies that the utilization of ANC four, SBA, and PNC among women with the richest households was 2.2 times, 3 times, and 3.4 times higher than in women with the poorest wealth quintile. Similar findings were also reported from complex measures of inequality. Both the absolute and relative complex measures of inequality indicated significant inequality in the utilization of maternal health services. For instance, the complex measures of inequality, PAR and PAF, were 25.1 and 56.7 for ANC four services, respectively.

A profound difference was reported between women who have attained higher education and those who have no education. The absolute measure of D for ANC four, SBA, and PNC was 42.1 pp., 56.7 pp., and 48.4 pp., respectively; likewise, the relative measure of R was 2.2 for ANC four, 2.4 for SBA, and 2.8 for PNC services. The absolute and relative measures of PAR and PAF were 31.6 and 71.4 in ANC four, and 36.2 and 58.8 in SBA. The simple and complex measures of inequality indicated the presence of a higher level of education-based inequality in the utilization of maternal health services.

The disparities in the utilization of maternal health services between urban and rural residents were reported through both absolute and relative measures of inequality. The value of D was 28.9 pp., 41.9 pp., and 31.9 pp. for ANC, SBA, and PNC services, respectively, while R was 1.8 for ANC four and SBA, and 1.9 for PNC services. Similarly, PAR was found to be 21.8, 31.6, and 24.1 for ANC four, SBA, and PNC services, respectively. The simple measure of D and the complex summary measure of inequality show the existence of a high level of residence-based inequality in the utilization of maternal health services, whereas the R indicates a moderate level of residence-based inequality in the use maternal services.

Our study also reported significant inequalities in the subnational regions of the inequality dimension. A simple measure of D indicates the coverage of ANC four and PNC in Addis Ababa city administration was higher by 38 pp. and 54.7 pp., respectively, compared with the SNNP. The simple measure of R also reported 2.1 for ANC four, 1.8 for SBA, 2.7 for PNC. Similarly, the complex measures PAR and PAF were reported for ANC four (28.1 and 63.5), SBA (37.7 and 61.2), and PNC (44.7 and 105.4). Both complex measures indicate significant inequalities in the utilization of maternal health services, suggesting that women from the Addis Ababa city administration receive better maternal health services compared to women from other regions.

A simple measure of D and R showed the existence of age-related inequalities in the utilization of maternal health services. The absolute measure of inequality D in SBA was −12.3 pp., suggesting a moderate level of age-related inequality favoring older women. The values of D for ANC four and PNC were − 4 pp. and − 5 pp., respectively, indicating low and moderate level of inequality. The relative measure of inequality R was 0.9 for ANC and PNC, and 0.8 for SBA, demonstrating a moderate level of age-related inequality. On the other hand, the complex measures of inequality, PAR and PAF, are both 0 for all maternal health indicators, suggesting no age-related equality in the utilization of maternal health services (Table 2).

**Table 2:** Level of inequality in the utilization of maternal health service based on summary measures in Ethiopia: evidence from PMA-Ethiopia, 2022.

		Antenatal care			Skilled birth attendant			Postnatal care		
Inequality dimension	Summary measures	Est	LB	UB	Est	LB	UB	Est	LB	UB
Mother’s age at birth	D	-4	−4.1	−3.9	−12.3	−12.4	−12.2	−5	−5.1	−4.9
	R	0.9	0.9	0.9	0.8	0.8	0.8	0.9	0.9	0.9
	PAR	0.0	−2.9	2.9	0.0	−2.8	−2.8	0.0	−2.9	2.9
	PAF	0.0	−6.6	6.6	0.0	−4.6	4.6	0.0	−6.8	6.8
Household wealth quintile	D	38.3	38.1	38.4	64.0	63.9	64.1	50.7	50.6	50.8
	R	2.2	2.2	2.2	3.0	3.0	3.0	3.4	3.3	3.4
	PAR	25.1	20.9	29.3	34.5	30.2	38.8	29.8	26.0	33.6
	PAF	56.7	47.2	66.2	56.1	49.1	63.1	70.2	61.2	79.2
Mother’s education level	D	42.1	42.0	42.2	56.7	56.6	56.8	48.4	48.3	48.5
	R	2.2	2.2	2.2	2.4	2.4	2.4	2.8	2.7	2.8
	PAR	31.6	28.5	34.8	36.2	32.9	39.5	33.6	30.5	36.6
	PAF	71.4	64.3	78.6	58.8	53.5	64.1	79.2	71.9	86.4
Place of residence	D	28.9	28.8	29.0	41.9	41.8	42.0	31.9	31.9	32.0
	R	1.8	1.8	1.8	1.8	1.8	1.8	1.9	1.9	1.9
	PAR	21.8	20.5	23.2	31.6	30.4	32.9	24.1	22.8	25.5
	PAF	49.3	46.3	52.3	58.8	53.5	64.1	56.8	53.6	60.0
Subnational region	D	38.0	37.8	38.2	43.1	43.0	43.2	54.7	54.6	54.8
	R	2.1	2.1	2.1	1.8	1.8	1.8	2.7	2.7	2.7
	PAR	28.1	24.1	32.2	37.7	35.6	39.8	44.7	40.7	48.7
	PAF	63.5	54.4	72.6	61.2	57.9	64.6	105.4	96.0	114.8

D, difference; R, ratio; PAR, population attributable risk; PAF, population attributable fraction; LB, lower bound; UB, upper bound; Est, point estimate.

*SNNP includes Central Ethiopia Regional State, South Ethiopia Regional State, and South West Ethiopia Peoples’ Region.

## Discussion

In this study, we examined the magnitude of socioeconomic and geographic related inequality in the utilization of maternal health services in Ethiopia. The coverage of maternal health utilization was 44.3, 61.5, and 42.4% for ANC four, SBA, and PNC services, respectively. The finding showed that the national coverage of the maternal indicators was below the national and WHO targets. To eliminate avoidable maternal deaths, the WHO recommends that countries attain over 90% coverage in three key maternal indicators (11, 15). The coverage of SBA and PNC services was higher than in the 2019 MEDHS report. However, the coverage of ANC four visits was similar to the MEDHS findings (16).

Findings from simple and complex measures of inequality confirmed the existence of a high level of socioeconomic and geographic related health inequality in the utilization of maternal health services across population subgroups in Ethiopia in 2022. The difference between the richest and the poorest wealth quintiles was 64 pp., 50.7 pp. and 38.3 pp. for the SBA, PNC, and ANC four services, respectively. Similarly, maternal health service utilization in the richest wealth quantile was 2.2, 3.0, and 3.4 times higher than in the poorest quantile in ANC four, SBA, and PNC services, respectively. This finding was consistent with a study conducted in Ethiopia and Ghana (42, 43). However, the difference between the poorest and richest groups was higher than in a study conducted in Indonesia and Nepal (34, 44). This difference can be explained by a variation in the study period, a difference in economic and social development, and the context of health care policies. The complex summary measures of inequality (PAR and PAF) showed the existence of significant inequality in the coverage of maternal health services. The value was higher in the three maternal health indicators, which implies an area of potential improvement in maternal health service coverage. For instance, PAR suggests that if the coverage of maternal health services in the four wealth quintiles (i.e., quintile 1, quintile 2, quintile 3, and quintile 4) were the same as the richest wealth quintile, there would be a potential increase of 25.1% for ANC four, 34.5% for SBA, and 29.8% for PNC service from the current national coverage. PAR is a useful measure to explain the contribution of within-country inequality to a country’s progress toward universal health coverage. The health service coverage gap indicates the proportion of health services that were required but not addressed, highlighting the additional coverage required to reach universal health coverage (4, 45).

**Figure 3:**
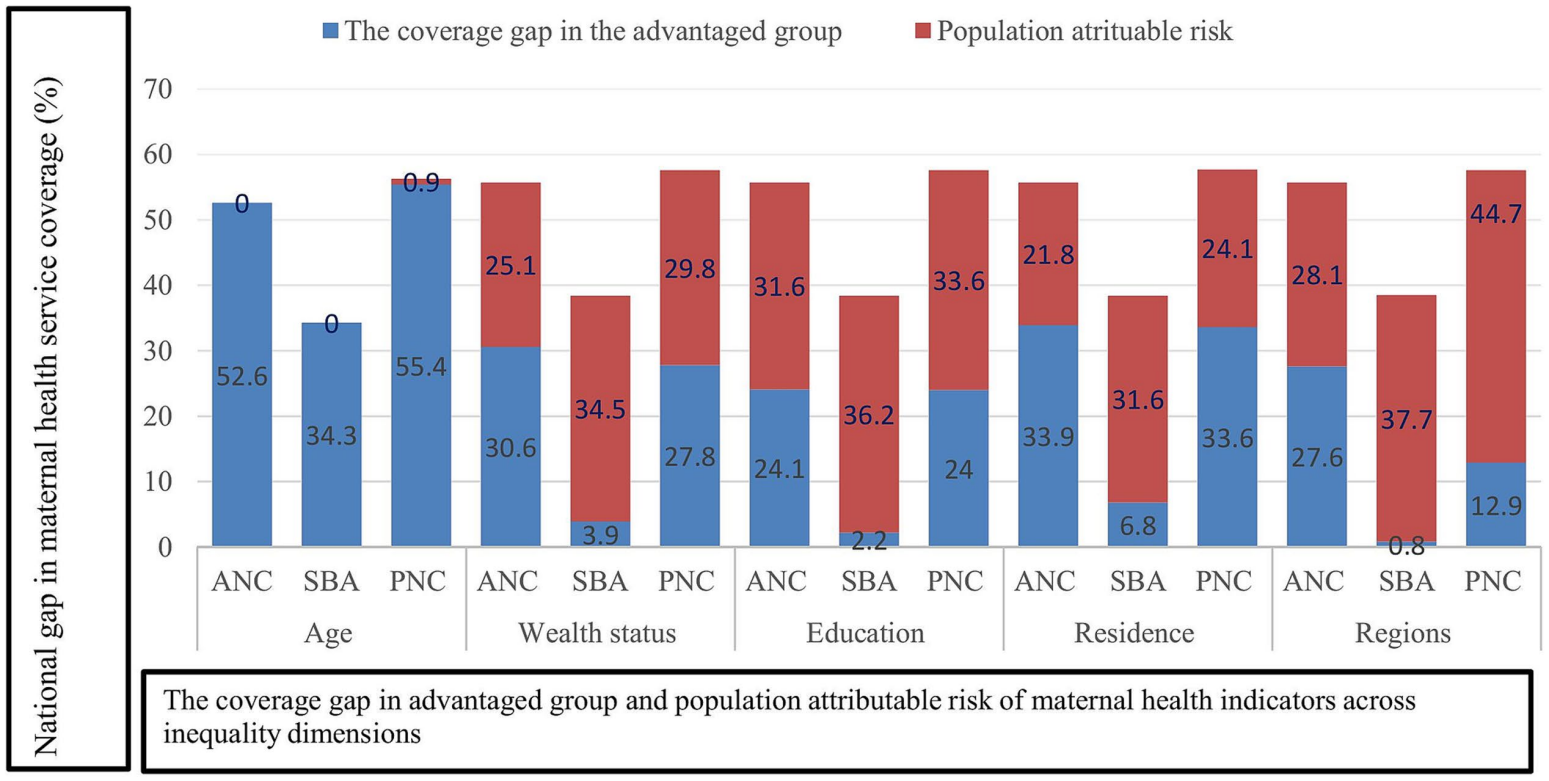
National average coverage gap in the maternal health service indicators by dimensions of inequality in Ethiopia, PMA 2022.

In our study, we illustrated the national coverage gap in all dimensions of inequality (age, wealth status, education, residence, and subnational regions) across the three maternal health indicators. Accordingly, the economic based national coverage gap was 57.6% in PNC, followed by 55.7% in ANC four, and 38.4% in SBA. A huge economic based national maternal health service coverage gap was observed in the three maternal health indicators (Figure 3). Generally, the findings of the study showed significant economic related health inequality in all three maternal health indicators, which means women from economically disadvantaged groups significantly lagged behind those with higher economic status in accessing maternal health services. Ethiopia has adopted a financial protection policy, including fee waiver systems, exemption services (for selected health services), and community based health insurance, to ensure access to healthcare services for its people without financial hardship (15). Maternal health services are offered free of charge to all women, regardless of their economic status, in Ethiopia. However, there is still a significant difference in the access to and use of maternal health services between the poorest and wealthiest population groups (46). Exempted user fees for maternal health services show promise in improving access to maternal health service utilization, but this initiative alone may not be adequate to ensure equitable access for all women to maternal health services (47). Explaining economic related inequality in maternal health service utilization is complex; both demand and supply-side factors can contribute to the inequality observed in accessing maternal health services (7–11). Women from poor households may face financial barriers to accessing maternal health services due to non-medical costs such as transportation and accommodation fees (48–51). Additionally, economically disadvantaged women might have a lower level of health-seeking behavior, and they are often compelled to prioritize income-generation activities to meet their household’s daily consumption needs (15, 48, 49). Furthermore, women from disadvantaged economic backgrounds may have a lower level of empowerment and restricted access to maternal health services (52, 53).

Our research demonstrated significant education related inequality in the utilization of maternal health services. There was a substantial difference of 42.1 pp. in the use of ANC four, 56.7 pp. in SBA, and 48.4 pp. in PNC services compared to women with no formal education. Also, the relative measure of R reported that in women with higher levels of education, maternal health service utilization was 2.2 times higher for ANC, 2.4 times higher for SBA, and 2.8 times higher for PNC compared to women with no formal education. The absolute measure D and relative measure R were both greater than 20 pp. and 2, suggesting the existence of a high level of inequality in the utilization of maternal health among the two population subgroups. This finding is similar to previous studies; high level of education related inequality was reported from the simple summary measures of health inequality (34, 42, 54). Like the simple summary measure of inequality, a significant high level of education related inequality in maternal health service utilization was also reported in the complex summary measures of inequality (PAR and PAF). For example, the value of PAR was higher in the three maternal health indicators, which implies an area of potential improvement in maternal health service coverage. If ANC four, SBA, and PNC service coverage for those with no education and a primary or secondary education level similar to that of a higher education level, the national coverage of ANC four, SBA, and PNC service could increase by 31.6, 36.2, and 33.6%, respectively, from the current level. Figure 3 depicts that the education based national coverage gap was higher in PNC (57.6%) and ANC four (55.7%). With the exception of age, the three maternal health indicators demonstrated a wide national coverage gap across all dimensions of inequality, including economic status, education level, place of residence, and subnational regions. This finding suggests that Ethiopia is far from achieving universal health service coverage in maternal health service indicators. Hence, effective interventions that address socioeconomic and geographic related inequality could greatly contribute to reducing the national maternal health service coverage gap (45). Overall, maternal health service utilization was higher among women with higher education levels. The possible explanation for this result could be that the higher the level of education attained, the higher the chances that women will change their attitude toward maternal healthcare utilization, including their maternal healthcare seeking behaviors (55). Additionally, they have relatively better access to and use of health information, and they can easily understand the benefits of maternal health services for women and newborns (56–58). On the other hand, higher education is also associated with wealth status. Women who attained higher education might have a better job and financial freedom to have a maternal health service (59, 60). Similarly, educated women have more autonomy and empowerment, allowing them to independently make decisions on the utilization of maternal health services (52, 53, 61).

In this study, urban rural health inequality was reported in the relative and absolute measures of inequality. In the present study, we found that urban women had a higher rate of utilizing ANC four, SBA, and PNC service by 28.9 pp., 41.9 pp., and 31.9 pp., respectively, compared to rural residents. Correspondingly, the utilization of ANC four and SBA services among urban women was 1.8 times higher than among rural women. Both absolute and relative measures of D and R imply the presence of significant inequality in the utilization of maternal health services between urban and rural women. The finding of this study were similar to those of previous studies carried out in Ethiopia (42), but this study was higher than a study conducted in Bangladesh (59), Armenia (62), Nepal (44), and Indonesia (34). Maternal health service utilization is lower among rural residents than urban women. The urban- rural difference in the utilization of maternal health services could be explained by distance to health facilities, lack of transportation, and inaccessibility to health facilities (50, 63). Additionally, women living in rural areas have limited access to education, limited empowerment, and reduced access to health information compared to women living in urban areas (64–66). Furthermore, differences in infrastructure, the unfair distribution of skilled healthcare providers, and the limited availability of medical supplies could contribute to the inequalities in the utilization of maternal health services between urban and rural residents (52, 63, 67). Similar to the simple summary measure of inequality, the complex summary measures of inequality, PAR and PAF, revealed that significant inequality was observed in the utilization of maternal health services between urban and rural resident women. The three maternal health indicators have the highest potential for improvement, as indicated by the PAR and PAF values. For instance, the value of PAR indicated that if the coverage of maternal health service indicators improved to the level of urban area, the national coverage of ANC four, SBA, and PNC services could be improved by 21.8, 31.6, and 24.1%, respectively, from the current level.

The present study also investigated the degree of inequality in the utilization of maternal health services across various regions of Ethiopia. Accordingly, a high level of inequality in the utilization of maternal health services was reported across different regions of Ethiopia. ANC four and PNC services had higher coverage in Addis Ababa city by 38 pp. and 54.7 pp., respectively, compared to the SNNP. In addition, the SBA had higher coverage in the Addis Ababa city by 43.1 pp. compared to Oromia region. The relative measure of R also confirmed the presence of a high level of inequality in ANC four and PNC services. The coverage of ANC four and PNC services in Addis Ababa city was 2.1 and 2.7 times higher than in the SNNP region, respectively, whereas the SBA coverage was 1.8 times higher in Addis Ababa city than in the Oromia region.

The complex summary measure of inequality PAR suggests that if the three regions achieved the same level of coverage for ANC four, SBA, and PNC services as Addis Ababa city, the national coverage of these services would increase by 28.1, 37.7, and 44.7%, respectively, compared to the present state. The variation in the utilization of maternal health services across the region could be explained by several factors such as variation in socioeconomic status, belief and cultural practices, as well as regional economic imbalance, infrastructure limitation, uneven budget distribution and health resources allocation, and variations in leadership quality across administrative regions (68).

The other dimension of inequality that we examined in our study was the age of the mother. Simple and complex summary measures of health inequality were not consistent across the three maternal health indicators. For example, the value of D demonstrated the presence of a low level of inequality between the younger and older age groups, whereas, in SBA, a moderate level of inequality was observed, which is lower by 12 pp. in older women. However, the complex measure of PAR and PAF was zero for ANC four, SBA, and PNC services. This indicates the absence of age-related inequality in the utilization of maternal health services. Moreover, if we were achieving equal coverage across all age groups, the national coverage of ANC four, SBA, and PNC services would remain unchanged. This study was similar to a study done in Indonesia (34).

### Policy implication

A high level of socioeconomic and geographic area-based inequality was observed in Ethiopia in the utilization of maternal health services. The findings of this study implied that inequality in the utilization of maternal health services is attributed to social determinants of health. The observed inequalities (measured difference) in the utilization of maternal health services represent health inequity since the difference is unfair, unjust and avoidable (7). The government and stakeholders should take action to reduce the substantial inequity observed in the utilization of maternal health services.

Policies, programs, and practices should prioritize the needs of subgroups that are falling behind. Enhanced measures are needed to address inequality in the utilization of maternal health services among socioeconomically disadvantaged subgroups and women from disadvantaged geographic areas. Reducing health inequality lies in implementing intersectoral and multidisciplinary strategies, including all sectors of the health system (56, 69). The strategies should also address both demand and supply side interventions (44, 52). Moreover, it is vital to strengthen healthcare system components such as human resources, commodities and supplies, health infrastructure, health information, financial protection mechanisms, and service delivery modalities (34, 44, 70). The government of Ethiopia is striving to address health inequality through a general approach that targets key social determinants of health, such as economic, educational, geographic, and gender-based disparities (18). However, solely relying on this approach might not swiftly reduce inequality among disadvantaged groups. Because it may take more than a decade to reduce economic, social, gender, and geographic differences within the population in developing countries (54, 56, 71). Thus, in efforts to address inequality in the utilization of maternal health services, it is important to take into account both general and specific approaches. Particularly, implementing targeted interventions for the most disadvantaged groups can help to reduce inequality in accessing maternal health services (71).

### Strengths and limitations

The strengths of this study include: Firstly, most prior studies relied on data from the Ethiopia Demographic and Health Survey to analyze inequality in maternal health services. However, using an additional or new source of data can help enrich the evidence. Hence, we used recent data from Performance Monitoring for Action Ethiopia (PMA Ethiopia). Secondly, we employed HEAT Plus software, which enables users to upload their own databases for measuring health inequalities. As a result, we prepared a new database using PMA data (other than the WHO database) and uploaded it to HEA Plus for analysis of inequality in the utilization of maternal health services. Finally, the study was collected among postpartum women so that it reduces recall bias. However, the study had the following limitations: The first limitation was that the study included women from three agrarian regions of Ethiopia (Amhara, Oromia, and SNNP) and from the city administrations (Addis Ababa). However, pastoralist regions, despite their smaller population representation, were not part of the study. This can have an effect on the findings. Secondly, the PMA data do not use direct measures of income, spending, or consumption. Therefore, we used an asset-based wealth index as a proxy measure for socioeconomic status.

## Conclusion

From the findings of the present study, we conclude that a high level of socioeconomic and geographic area related inequality exists in the utilization of ANC four, SBA, and PNC services in Ethiopia. Women from socioeconomically disadvantaged subgroups and women from disadvantaged geographic areas significantly lagged behind in the utilization of maternal health services. This study also highlighted that the three maternal health indicators have a higher potential for improvement in all dimensions of inequality, with the exception of age. The observed inequality in this study reflects inequity in the use of maternal health services. This disparity is unfair, unjust, and avoidable through the right mix of policy interventions. Therefore, implementing targeted interventions for the most disadvantaged groups can help to reduce inequality in accessing maternal health services and improve the national coverage of maternal health indicators.

## Data Availability

Publicly available datasets were used for this study. To access the data, we completed an online registration process and submitted requests to the PMA data manager. Upon receiving approval, we downloaded the dataset from the PMA online archive at www.pmadata.org.
